# Response inhibition is modulated by functional cerebral asymmetries for facial expression perception

**DOI:** 10.3389/fpsyg.2013.00879

**Published:** 2013-11-22

**Authors:** Sebastian Ocklenburg, Vanessa Ness, Onur Güntürkün, Boris Suchan, Christian Beste

**Affiliations:** ^1^Biopsychology, Institute of Cognitive Neuroscience, Faculty of Psychology, Ruhr-University BochumBochum, Germany; ^2^Neuropsychology, Institute of Cognitive Neuroscience, Faculty of Psychology, Ruhr-University BochumBochum, Germany; ^3^Cognitive Neurophysiology, Department of Child and Adolescent Psychiatry, University of DresdenDresden, Germany

**Keywords:** executive functions, Go/Nogo task, EEG, ERP, laterality, lateralization, Nogo-N2, Nogo-P3

## Abstract

The efficacy of executive functions is critically modulated by information processing in earlier cognitive stages. For example, initial processing of verbal stimuli in the language-dominant left-hemisphere leads to more efficient response inhibition than initial processing of verbal stimuli in the non-dominant right hemisphere. However, it is unclear whether this organizational principle is specific for the language system, or a general principle that also applies to other types of lateralized cognition. To answer this question, we investigated the neurophysiological correlates of early attentional processes, facial expression perception and response inhibition during tachistoscopic presentation of facial “Go” and “Nogo” stimuli in the left and the right visual field (RVF). Participants committed fewer false alarms after Nogo-stimulus presentation in the left compared to the RVF. This right-hemispheric asymmetry on the behavioral level was also reflected in the neurophysiological correlates of face perception, specifically in a right-sided asymmetry in the N170 amplitude. Moreover, the right-hemispheric dominance for facial expression processing also affected event-related potentials typically related to response inhibition, namely the Nogo-N2 and Nogo-P3. These findings show that an effect of hemispheric asymmetries in early information processing on the efficacy of higher cognitive functions is not limited to left-hemispheric language functions, but can be generalized to predominantly right-hemispheric functions.

## INTRODUCTION

Intentional response inhibition is an executive control mechanism that is mainly regulated by the prefrontal cortex (e.g., Chikazoe, 2010). A commonly used method to experimentally assess this cognitive function is the Go/Nogo task in which participants have to perform a simple motor action (e.g., pressing a key on a PC keyboard) in response to one type of stimulus (Go), while they have to refrain from responding when the other type of stimulus (Nogo) is presented (e.g., Beste et al., 2010, 2013). One important factor modulating performance in Go/Nogo tasks is bottom-up information processing of the used stimuli (Knudsen, 2007), and it has been shown that hemispheric asymmetries for the used stimulus material affect the efficacy of response inhibition. For instance, Ocklenburg et al. (2011) tachistoscopically presented verbal “Go” and “Nogo” stimuli in the left and the right visual field (RVF) and reported that participants committed fewer false alarms when reacting to verbal Nogo-stimuli presented in the RVF than to stimuli presented in the left visual field (LVF), reflecting the well-known left-hemispheric dominance for processing of verbal stimuli (Hugdahl, 2000; Corballis, 2012; Hirnstein et al., 2012; Ocklenburg et al., 2012; Bless et al., 2013; Cai et al., 2013; Ocklenburg et al., 2013). Thus, initial stimulus representation in the non-dominant hemisphere seems to be leading to a less efficient inhibition process, an idea that was also supported by another divided visual field Go/Nogo study with verbal stimuli (Measso and Zaidel, 1990). However, it is unclear whether this effect is specific for the language system or a general principle that also applies to other types of lateralized cognition. Therefore, it was the aim of the present study to investigate whether the efficacy of response inhibition processes is also modulated by a typical right-sided functional asymmetry, the well-known right-hemispheric dominance for face processing (Levine et al., 1988; Rossion et al., 2003; Dien, 2009; Sung et al., 2011; Gainotti, 2013). To this end, we recorded event-related potentials (ERP’s) during tachistoscopic presentation of facial “Go” and “Nogo” stimuli in the LVF and RVF.

The earliest ERP component that was assessed was the P1, a positive component with a peak between 80 to 120 ms after stimulus presentation (Proverbio et al., 2012) which is centered over the occipital cortex (electrodes O1 and O2). The P1 is the earliest endogenous visual ERP component and is reliably elicited in response to visual stimuli (Taylor, 2002; de Haan et al., 2003). It has been shown to be modulated by a number of factors, including stimulus characteristics and attentional processes (Herrmann and Knight, 2001; Herrmann et al., 2005; Beste et al., 2008; Martin et al., 2008; Wild-Wall et al., 2012). Interestingly, the P1 has been suggested to reflect early face processing (Itier and Taylor, 2002) and it has been found that the P1 is shorter to faces than inverted faces (Taylor, 2002) and that for central stimulus presentation, P1 amplitudes are more positive after presentation of stimuli showing make-up resembling a human face compared to animal-like make-up (Luo et al., 2013). However, there are also studies that did not find any effect of faces compared to non-face visual patterns on the P1 (e.g., see Rossion et al., 1999). Findings regarding lateralization of the P1 are ambiguous, with some work reporting no significant side effects (Herrmann et al., 2005) while a recent study by Proverbio et al. (2012) reported that the P1 in a face-sex categorization task was left lateralized in women and bilateral in men.

The second early ERP component that was assessed was the N170 (Bentin et al., 1996; Itier et al., 2006, 2011). The N170 is a negative component which peaks about 130 to 170 ms after stimulus presentation, is usually centered over the occipito-temporal cortex (Bentin et al., 1996; Eimer, 2000; Rossion and Gauthier, 2002; Rossion et al., 2003; Bieniek et al., 2013). For central stimulus presentation, N170 amplitudes are more negative after presentation of face-like make-up stimuli compared to animal-like make-up stimuli (Luo et al., 2013). Functionally, it is thought to reflect structural encoding of faces (Herrmann et al., 2005). Rossion et al. (2003) reported right lateralization of the N170 for faces. In contrast, it was bilateral for cars and left-lateralized for words. In accordance with these findings, right lateralization of the N170 was also reported by several other studies (e.g., Bentin et al., 1996; Balconi and Lucchiari, 2005; Maurer et al., 2008; Mercure et al., 2008; but see: Proverbio et al., 2010).

In addition to these early ERP components, it is also of interest to assess whether the neurophysiological correlates of response inhibition, such as the Nogo-N2 and Nogo-P3, are modulated by tachistoscopic presentation of facial Go and Nogo stimuli. This is particularly interesting in order to elucidate whether lateralized processing in perceptual and early attentional cognitive processes affect higher cognitive functions such as executive control. The Nogo-N2 is a negative component that is thought to be related to either pre-motor inhibition (Falkenstein et al., 1999) or response conflict (Nieuwenhuis et al., 2003). Ocklenburg et al. (2011) could show that the N2 is lateralized when verbal “Go” and “Nogo” stimuli are presented tachistoscopically in the left and the RVF, so that initial stimulus processing is limited to one hemisphere. In accordance with the conflict hypothesis by Nieuwenhuis et al. (2003), the Nogo-N2 was stronger in response to Nogo-stimuli presented in the LVF. Thus, initial stimulus processing by the subdominant hemisphere leads to a stronger response conflict than initial processing by the dominant hemisphere, even if the inhibition process itself is driven by bilateral prefrontal networks. Apart from the Nogo-N2, the Nogo-P3 has also been related to response inhibition. The Nogo-P3 is a late positive component that has been linked to the evaluation of successful inhibition (Band and van Boxtel, 1999; Roche et al., 2005; Sehlmeyer et al., 2010; Smith et al., 2010, 2013; Beste et al., 2011a). For the Nogo-P3, Ocklenburg et al. (2011) did not observe as clear an asymmetry effect as for the Nogo-N2, but there was a non-significant trend for lateralization on Nogo-trials only.

Based on these findings, we hypothesize that in our task, participants should commit fewer false alarms on Nogo-trials after stimulus presentation in the LVF. This behavioral performance asymmetry should be accompanied by electrophysiological asymmetries on the level of the P1, N170, N2 and possibly P3.

## MATERIALS AND METHODS

### PARTICIPANTS

Twenty-eight neurologically healthy volunteers (17 female, 11 male) with a mean age of 24.35 years (range: 21–32 years) participated in the present study. Handedness was assessed using the German version of the Edinburgh Handedness Inventory (EHI; Oldfield, 1971). All participants were right-handed according to the results of EHI (mean laterality quotient 91.5; range 56–100). All participants gave written informed consent and were treated in accordance with the declaration of Helsinki. The study was approved by the ethics committee of the Faculty of Psychology, Ruhr-University Bochum, Germany.

### EXPERIMENTAL PARADIGM

A Go/Nogo task was used to measure response inhibition to face stimuli that were presented tachistoscopically on a 17^′′^ CRT computer monitor. Subjects had to react to “Go”-stimuli by pressing a key on a custom-made reaction-pad, and to refrain from pressing the key after a “Nogo”-stimulus was presented. The stimuli were two morphed male faces taken from the BESST (Bochum Emotional Stimulus Set; Thoma et al., 2012): a friendly and an angry face. To control for possible valence effects of the emotional faces, each participant completed two blocks in randomized order, one block in which the friendly face was the “Go”-stimulus and the angry face was the “Nogo”-stimulus and another block in which the angry face was the “Go”-stimulus and the friendly face was the “Nogo”-stimulus. On half of the trials within each experimental block, subjects responded toward the “GO” stimulus with the index finger of their right hand, and on the other half they responded with their left index finger toward the “GO” stimulus, in randomized order. Overall, the task consisted of 2560 trials (1280 per block), with 1792 trials being “Go”-trials (70%) and 768 trials being “Nogo” trials (30%). On half of the trials, stimuli were presented in the LVF, in the other half in the RVF, in randomized order. At the beginning of the experiment, participants were instructed to place the head on a chin rest placed at a distance of 57 cm from the monitor. Accordingly, 1 cm on the screen represented 1^°^ of visual angle. Stimuli had a maximum width of 3^°^ visual angle (from ear to ear) and a maximum height of 5^°^ visual angle (from the neck to the top of the head.). Subjects were instructed to fixate a black fixation cross that was presented in the middle of the screen throughout the experiment. Each trial started with tachistoscopic presentation of the stimulus for 185 ms. Afterward, the central fixation cross was presented for 365 ms (Ocklenburg et al., 2011). The inter-trial interval was randomized between 750 and 950 ms. Only the central fixation cross was presented during this interval.

### EEG RECORDING AND ANALYSIS

EEG data were recorded from 65 active Ag–AgCl electrodes at standard scalp positions against a reference electrode located at FCz. Data were recorded with a sampling rate of 1000 Hz, and down-sampled off-line to 128 Hz. All electrode impedances were kept below 5 kΩ. The data was band-pass filtered (0.5–20 Hz) offline before further data processing and then visually inspected to remove technical artifacts. Horizontal and vertical eye movements as well as pulse artifacts were then corrected using an independent component analysis (ICA; Infomax algorithm) applied to the un-epoched data set. In the epoched data, automated artifact rejection procedures with the following rejection criteria were applied: maximum voltage steps of more than 50 μV/ms, maximal value differences of 200 μV in a 200 ms interval, or activity below 0.1 μV. To achieve a reference-free evaluation, peak, and latency analyses were performed after calculation of current source density (CSD) of the signals (Perrin et al., 1989). For statistical analysis, amplitudes were quantified relative to a baseline covering 200 ms before stimulus presentation until stimulus onset. Averaging was locked at the time point of “Go”- or “Nogo”-stimulus presentation and analysis epochs had a length of 1500 ms (from 200 ms before stimulus presentation until 1300 ms after stimulus presentation). Subsequent to averaging, P1, N170, and N2 amplitudes in “Go”- and “Nogo”-trials were calculated relative to baseline using only trials on which participants had reacted correctly. P3 amplitudes were calculated relative to N2 amplitudes. For each ERP component, the local maximum (for positive components) or minimum (for negative components) within a given time window (P1: 50–150 ms after stimulus presentation; N170: 100–200 ms; N2: 200–400 ms; P3: 250–550 ms) was determined. This was done using a semi-automated search function implemented in the analysis software. The results of the automated search were then visually inspected and corrected of necessary. For the P1, amplitudes and latencies were quantified at the standard positions O1 and O2, while for the N170, amplitudes and latencies were quantified at electrodes CP5 and CP6. For the Nogo-N2 and Nogo-P3, amplitudes and latencies were quantified at the standard position FCz.

### STATISTICAL ANALYSIS

The behavioral data (i.e., rate of false alarms on Nogo trials as well as misses and reaction times on Go-trials) were analyzed using paired samples t-tests to compare performance after stimulus presentation in the LVF and RVF. P1 and N170 data were analyzed using repeated measures analyses of variance (ANOVAs) with the within-subjects factors electrode (P1: O1 and O2; N170: CP5 and CP6), condition (Go, Nogo), and visual half-field (RVF, LVF). N2 and P3 data were analyzed using repeated measures ANOVAs with the within-subjects factors condition (Go, Nogo) and visual half-field (RVF, LVF). When appropriate, the degrees of freedom were adjusted using Greenhouse–Geisser correction. The *p*-levels for *post hoc* testing were adjusted using Bonferroni correction. Effect sizes are provided as the proportion of variance accounted for (partial η^2^). As a measure of variability, the standard error of the mean (SEM) was used. All statistical analyses were conducted using IBM SPSS Statistics 20.

## RESULTS

### BEHAVIORAL DATA

In Nogo-trials, the false alarm rate was higher for stimuli that were presented in the RVF (29.82% ± 3.77) than for stimuli that were presented in the LVF (25.69% ± 3.12; *t*_(27)_ = 2.39; *p* < 0.05). In contrast, no visual field difference was observed for the number of misses on Go-trials (RVF: 8.43% ± 2.16; LVF: 8.43% ± 2.29; *t*_(27)_ = 0.01; *p* = 0.99) or reaction time on correct Go-trials (RVF: 472.09 ms ± 11.19; LVF: 468.11 ms ± 11.22; *t*_(27)_ = -1.14; *p* = 0.27).

### NEUROPHYSIOLOGICAL DATA

#### P1

**FIGURE 1:**
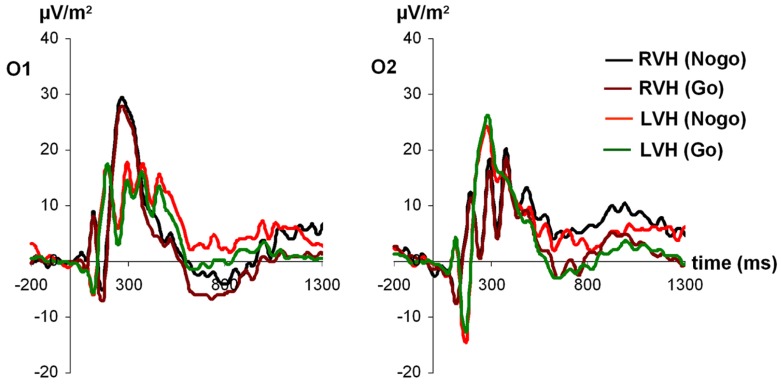
**Time course of ERP components at electrodes O1 and O2 in the Go and Nogo condition after stimulus presentation in the left and right visual field.** Time point 0 indicates the point of Go- or Nogo-stimulus presentation.

For P1 amplitudes (see **Figure 1**), the ANOVA revealed a main effect of electrode [*F*_(1,27)_ = 4.42; *p* < 0.05; partial η^2^= 0.14], indicating a more positive amplitude of the P1 at the left-sided electrode O1 (24.47 ± 2.94) compared to the right-sided electrode O2 (18.86 ± 3.07). In addition, a significant interaction visual half-field × electrode [*F*_(1,27)_ = 4.38; *p* < 0.05; partial η^2^= 0.14] indicated that after stimulus presentation in the RVF, the P1 was more positive at the left-sided electrode O1 (27.71 ± 4.08) than at the right-sided electrode O2 (16.01 ± 2.59; Bonferroni corrected *post hoc* test: *p* < 0.01). In contrast, after stimulus presentation in the LVF, no amplitude difference between the two electrodes was observed (O1: 21.22 ± 2.62; O2: 21.71 ± 4.12; Bonferroni corrected *post hoc* test: *p* = 1.00). Moreover, a significant interaction visual half-field × condition emerged [*F*_(1,27)_ = 4.35; *p* < 0.05; partial η^2^= 0.14], indicating a visual half-field difference between Go- and Nogo-trials, but both *post hoc* tests failed to reach significance, indicating a rather weak effect (Go-trials: LVF: 21.40 ± 2.67; RVF: 20.40 ± 3.05; Bonferroni corrected *post hoc* test: *p* = 1.00; Nogo-trials: LVF: 21.53 ± 2.86; RVF: 23.32 ± 2.88; Bonferroni corrected *post hoc* test: *p* = 0.74). All other main effects and interactions failed to reach significance (all *p* > 0.11).

For P1 latencies, only the visual half-field × condition interaction reached significance [*F*_(1,27)_ = 4.37; *p* < 0.05; partial η^2^= 0.14], indicating a trend toward a smaller P1 latency on Nogo-trials after stimulus presentation in the LVF (126.27 ms ± 9.20) compared to the RVF (RVF: 139.23 ms ± 8.79; Bonferroni corrected *post hoc* test: *p* = 0.33; Go-trials: LVF: 140.49 ms ± 9.96; RVF: 132.95 ms ± 10.30; Bonferroni corrected *post hoc* test: *p* = 0.80). However, since both *post hoc* tests failed to reach significance, this effect seems to be rather weak. Moreover, a trend toward a visual half-field × electrode interaction emerged [*F*_(1,27)_ = 3.46; *p* = 0.07; partial η^2^= 0.11]. All other main effects and interactions failed to reach significance (all *p* > 0.11).

#### N170

**FIGURE 2:**
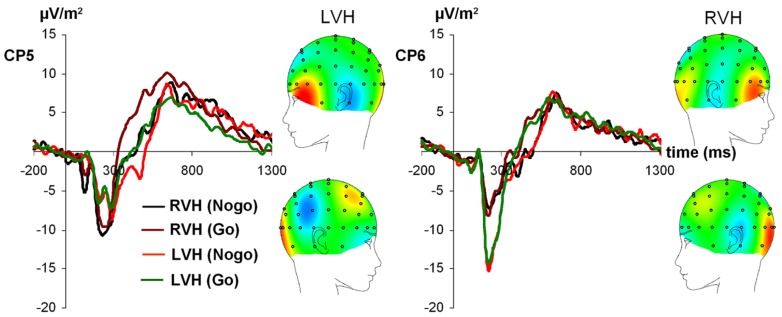
**Time course of ERP components at electrodes CP5 and CP6 in the Go and Nogo condition after stimulus presentation in the left and right visual field.** Time point 0 indicates the point of Go- or Nogo-stimulus presentation.

For N170 amplitudes (see **Figure 2**), the ANOVA revealed a significant main effect of condition [*F*_(1,27)_ = 4.48; *p* < 0.05; partial η^2^= 0.14], indicating that the N170 was more negative on Nogo-trials (-15.76 ± 1.20) compared to Go-trials (-14.34 ± 1.12). Moreover, an interaction visual half-field × electrode emerged [*F*_(1,27)_ = 17.08; *p* < 0.001; partial η^2^= 0.39], indicating that after presentation of a face in the LVF, the N170 was more negative at the right-sided electrode CP6 [-19.03 ± 1.87] than at the left-sided electrode CP5 (-12.68 ± 1.56, Bonferroni-corrected *post hoc* test: *p* < 0.05]. For presentation of a face in the RVF, a trend toward the opposite direction was observed (CP5: -16.05 ± 1.71; CP6: -12.45 ± 1.35), but the *post hoc* test failed to reach significance (*p* = 0.19). In addition, a trend toward a condition × visual half-field × electrode emerged [*F*_(1,27)_ = 2.88; *p* = 0.10; partial η^2^= 0.10]. All other main effects and interactions failed to reach significance (all *p* > 0.13). The visual half-field × electrode interaction also reached significance for N170 latency [*F*_(1,27)_ = 6.25; *p* < 0.05; partial η^2^= 0.19]. After presentation of a face in the LVF, the N170 had a longer latency at the right-sided electrode CP6 (173.55 ms ± 5.14) than at the left-sided electrode CP5 (147.18 ms ± 9.48, Bonferroni-corrected *post hoc* test: *p* < 0.05). For presentation of a face in the RVF, no significant difference between electrodes was observed (CP5: 173.97 ms ± 9.13; CP6: 165.46 ms ± 8.08; Bonferroni-corrected *post hoc* test: *p* = 0.80). All other main effects and interactions failed to reach significance (all *p* > 0.11).

#### N2 and P3

**FIGURE 3:**
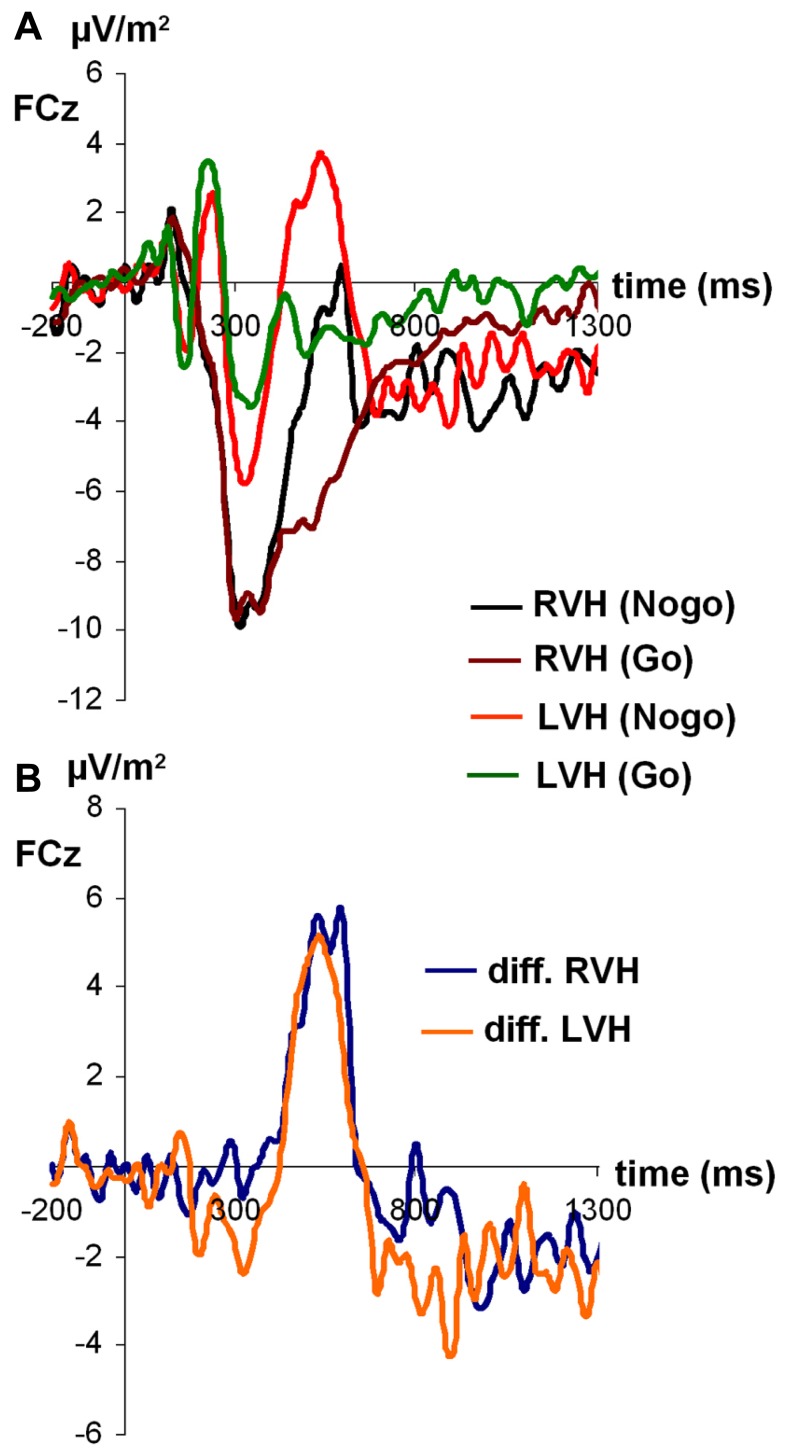
**(A)** Time course of ERP components at electrodes FCz in the Go and Nogo condition after stimulus presentation in the left and right visual field. **(B)** Time course of ERP components at electrodes FCz as difference between Go and Nogo condition for the left and right visual field. Time point 0 indicates the point of Go- or Nogo-stimulus presentation.

For N2 amplitudes (see **Figure 3**), the ANOVA revealed a significant main effect of condition [*F*_(1,27)_ = 6.45; *p* < 0.05; partial η^2^= 0.19), indicating that the N2 was more negative on Nogo-trials (-14.06 ± 1.71) than on Go-trials (-10.57 ± 1.36). Moreover, a significant main effect of visual half-field emerged [*F*_(1,27)_ = 4.91; *p* < 0.05; partial η^2^= 0.15], indicating that the N2 was more negative after stimulus presentation in the RVF (-14.25 ± 1.91) than after stimulus presentation in the LVF (-10.37 ± 1.32). The visual half-field × condition interaction failed to reach significance (*p* = 0.36). For N2 latencies, all effects failed to reach significance (all *p* > 0.11).

Due to N2 amplitude differences, P3 amplitudes were not determined peak-to-baseline but peak-to-peak, with the N2 serving as baseline. Only the main effect of condition reached significance [*F*_(1,27)_ = 13.99; *p* < 0.05; partial η^2^= 0.34], indicating that Δ was larger on Nogo-trials (23.50 ± 2.43) than on Go-trials (15.21 ± 1.96). All other effects failed to reach significance (all *p* > 0.20).

For P3 latencies, the main effect of visual half-field reached significance [*F*_(1,27)_ = 6.07; *p* < 0.05; partial η^2^ = 0.18], indicating that the P3 had a longer latency after stimulus presentation in the RVF (492.89 ms ± 20.11) than after stimulus presentation in the LVF (410.30 ms ± 27.31). This effect was modulated by condition, as indicated by a significant interaction visual half-field × condition [*F*_(1,27)_ = 5.99; *p* < 0.05; partial η^2^ = 0.18]. Interestingly, the visual half-field difference reached significance only on Nogo-trials (LVF: 399.28 ± 31.14; RVF: 551.89 ± 26.38; Bonferroni-corrected *post hoc* test: *p* < 0.01), but not on Go-trials (LVF: 421.32 ± 38.99; RVF: 433.87 ± 33.59; Bonferroni-corrected *post hoc* test: *p* = 1.00). The main effect of condition failed to reach significance (*p* = 0.18).

Since there is some controversy surrounding the use of the peak amplitude as a measure for the P3 (Luck, 2005), we also calculated the mean amplitude from 400 to 500 ms after stimulus presentation as an alternative measure for the P3. As for the peak amplitude, the main effect of condition reached significance [*F*_(1,27)_ = 10.95; *p* < 0.01; partial η^2^ = 0.29], indicating the P3 was more positive on Nogo-trials (1.21 ± 2.32) than on Go-trials (-4.18 ± 1.51). Moreover, the main effect of visual half-field reached significance [*F*_(1,27)_ = 6.90; *p* < 0.05; partial η^2^ = 0.20], indicating a more positive P3 after stimulus presentation in the LVF (1.71 ± 1.96) than after stimulus presentation in the RVH (-4,68 ± 2.33). The interaction failed to reach significance (*p* = 0.09).

## DISCUSSION

Functional cerebral asymmetries have been shown to modulate the efficacy of executive functions (Measso and Zaidel, 1990; Ocklenburg et al., 2013). While previous studies investigated how the left-hemispheric language dominance affects these prefrontally mediated cognitive functions, the present study was aimed at answering the question how executive functions are modulated by the right-hemispheric dominance for face processing. To this end, we recorded ERPs on a tachistoscopic version of the classic Go/Nogo task in which faces were presented in the left and RVF.

Behaviorally, participants committed fewer false alarms on Nogo-trials after stimulus presentation in the LVF. In line with the results of several earlier studies using the divided visual field technique with face stimuli (Leehey and Cahn, 1979; Young and Bion, 1981; Levine and Koch-Weser, 1982; Young, 1984; Young et al., 1985;Gainotti, 2013), this finding indicates greater efficacy of the right hemisphere for facial expression perception. In contrast, no hemispheric asymmetries were observed for accuracy or reaction times on Go-trials, which may be attributed to low task demands in the Go-condition possibly resulting in a ceiling effect. Moreover, this finding is also in line with the behavioral results of earlier studies that used divided visual fields versions of the Go/Nogo Task with verbal stimuli. These studies found that response inhibition is more efficient when initial stimulus processing is performed by the dominant hemisphere (Measso and Zaidel, 1990; Ocklenburg et al., 2013). Our findings indicate that this connection between functional hemispheric asymmetries and executive functions is not limited to left-hemispheric language function, but can also be observed for right-hemispheric functions.

In the ERP data, asymmetries were observed for various components in different cognitive processing stages. In accordance with the results of Proverbio et al. (2012) for female participants, we found left lateralization of the P1 after stimulus presentation in the RVF. Stimulus presentation in the LVF, however, did not lead to any asymmetry effects. This finding further supports the assumption of Proverbio et al. (2012) that for some types of face-processing tasks at least some left-hemispheric functions are necessary. Specifically, Proverbio et al. (2012) argued that facial tasks which require a high amount of local feature analyses may lead to left-lateralization of the P1 because local visual analyses are known to activate more left-hemispheric networks than global visual analyses (e.g., Hellige, 1996; Yovel et al., 2001). Since we used emotional faces in the present study, which differed mainly with regard to those parts of the face that communicate emotions (e.g., mouth, eyes, and eye-brows), one could speculate that participants partly relied on local visual analysis of these face features to react correctly, ultimately leading to the observed left-lateralization of the P1.

For the N170, the largest negative amplitude was observed at the right-sided electrode CP6 after stimulus presentation in the LVF. Moreover, only after stimulus presentation in the LVF (and thus initial stimulus processing in the dominant right hemisphere), did a significant amplitude difference between right- and left-sided electrodes emerge. Here, the N170 had a more negative amplitude at the right compared to the left electrode site. After stimulus presentation in the RVF (and thus initial stimulus processing in the non-dominant left hemisphere), no electrode difference was observed. Thus, in line with other studies reporting right-sided lateralization of the N170 (e.g., Bentin et al., 1996; Rossion et al., 2003; Balconi and Lucchiari, 2005; Maurer et al., 2008; Mercure et al., 2008), our results further support the assumption that the N170 is specifically driven by right-hemispheric brain areas, e.g., the fusiform gyrus or the superior temporal sulcus (Schweinberger et al., 2002; Shibata et al., 2002; Dalrymple et al., 2011). In contrast to the clear right-lateralization of the N170 amplitudes, the N170 had a longer latency over the right than over the left hemisphere when a stimulus was presented in the LVF. Interestingly, a similar finding has also been reported by Proverbio et al. (2012) for central stimulus presentation. In line with the discussion of the P1 findings, this result could be indicative of a need for left-hemispheric processing for certain aspects of our task, e.g., a local feature analysis of the emotional content of the face.

In general, the Nogo-N2 and Nogo-P3 data indicated that our tachistoscopic divided visual field version of the Go/Nogo task worked as intended, since we observed the typical pattern of results for these components. In accordance with previous studies utilizing this paradigm with central stimulus presentation (Beste et al., 2011b; Smith and Douglas, 2011), the Nogo-N2 was more negative after Nogo- than after Go-stimuli, and the Nogo-P3 was more positive after Nogo- than after Go-stimuli. For central stimulus presentation, both the Nogo-N2 and the Nogo-P3 are focused over fronto-central electrode sites (Falkenstein, 2006; Beste et al., 2010) and their generators have been localized mainly in the orbitofrontal cortex (Beste et al., 2010), with some authors reporting a right-shifted topography (Falkenstein, 2006), the inferior frontal cortex (Aron et al., 2004), and the anterior cingulate cortex (Nieuwenhuis et al., 2003).

In contrast to Ocklenburg et al. (2011) who found that the Nogo-N2 was stronger in response to Nogo-stimuli initially processed by the subdominant hemisphere, we found that for facial stimuli, the Nogo-N2 was more negative after initial processing in the subdominant hemisphere, regardless of condition. This difference between the two studies could possible indicate a response conflict in the Go-condition in the present study. For example, the higher stimulus complexity in the present paradigm could have rendered it more difficult for participants to react correctly on both Go and Nogo-trials than in the study by Ocklenburg et al. (2011). This assumption is supported by false alarm rates being overall higher in the present study than in studies using verbal stimuli (present study: RVF: 29.82%; LVF: 25.69%; Measso and Zaidel, 1990: RVF: 11.8%; LVF: 20.1%; Ocklenburg et al., 2011: RVF: 12.9%; LVF: 16.4%). Moreover, the miss rate for go-stimuli was around 8% in the present study, indicating that even when being asked to execute the predominant go-reaction, participants sometimes experienced problems to perform correctly. In addition to the generally higher complexity of the facial stimuli used in the present study, verbal stimuli typically used in Go/Nogo tasks (e.g., the words “Press” and “Stop”) are usually highly overlearned, since they have been associated with performing a reaction or refraining from doing so in everyday life. In contrast, in the present study, participants had to learn which stimuli represented a Go-signal or Nogo-signal during the experiment.

In addition to the Nogo-N2 results, we also observed an effect of functional cerebral asymmetries for facial expression perception on Nogo-P3 latencies. On Nogo-, but not on Go-trials, the P3 had a longer latency if initial stimulus processing was conducted by the non-dominant left hemisphere. Thus, initial stimulus processing by the dominant right hemisphere leads to faster evaluation of the inhibition process (Band and van Boxtel, 1999; Roche et al., 2005; Smith et al., 2010, 2013).

There are a few methodological considerations that have to be taken into account when interpreting the present ERP results. First of all, the P1 effects seem to be rather weak, since the half-field × condition interaction reached significance for both amplitudes and latencies, but both *post hoc* tests failed to reach significance for both variables after Bonferroni correction. This potential issue might be due to the fact that the P1 is not specifically elicited by perception of faces, but by perception of visual stimuli in general (Taylor, 2002; de Haan et al., 2003). To address this potential lack of statistical power to reliably detect P1 asymmetry effects, future studies investigating this topic should test larger samples and use a higher number of trials than the present work. One methodological consideration that has to be taken into account when interpreting the N2 and P3 results is the fact that it is not clear to what extent results obtained in a paradigm with lateralized stimulus presentation allow to draw conclusions about the impact of hemispheric asymmetries when stimuli are presented in the center of the visual field. For example, ERP studies in the field of hemispheric asymmetries in global vs. local processing demonstrate that central vs. lateralized presentation could affect the occurrence of hemispheric asymmetries: while all ERP studies with central stimulus presentation reported hemispheric asymmetries, some studies with laterally presented stimuli failed to replicate this finding (see Volberg and Hübner, 2004, for an overview). Thus, it would be interesting for futures studies investigating the impact of lateralization on executive functions to include a condition with central stimulus presentation in addition to stimulus presentation in the LVH and RVF. In regard to the present results, this would allow to differentiate hemispheric asymmetries for centrally presented faces (e.g., as reported by Rossion et al., 2003, for the N170) from hemispheric asymmetries following laterally presented stimuli.

Taken together, the present findings show that hemispheric asymmetries in information processing as reflected by early ERP components such as the N170 affect behavioral performance indicators as well as neurophysiological correlates of higher cognitive functions. In principle, initial stimulus processing by the dominant hemisphere leads to more efficient execution of subsequent cognitive tasks, even if task-related ERP components are mediated by bilateral neuronal networks, as is the case for Nogo-N2 and Nogo-P3 (Ocklenburg et al., 2011). This principle is not limited to left-hemispheric language functions, as has been suggested by previous studies, but can also be applied to predominantly right-hemispheric functions. However, it is obvious that the results for facial stimuli do not completely mirror the results for verbal stimuli. Thus, the present findings also indicate that it is important to consider both the specific neurobiological properties of the involved cognitive system as well as stimulus variables such as complexity when investigating the impact of functional cerebral asymmetries in information processing on higher cognitive systems.

## Conflict of Interest Statement

The authors declare that the research was conducted in the absence of any commercial or financial relationships that could be construed as a potential conflict of interest.
